# Mass Production of the Black Soldier Fly, *Hermetia illucens* (L.), (Diptera: Stratiomyidae) Reared on Three Manure Types

**DOI:** 10.3390/ani10071243

**Published:** 2020-07-21

**Authors:** Chelsea D. Miranda, Jonathan A. Cammack, Jeffery K. Tomberlin

**Affiliations:** 1 EVO Conversion Systems, LLC, College Station, TX 77845, USA; jacammack@evoconsys.com; 2 Department of Entomology, Texas A&M University, College Station, TX 77843, USA; jktomberlin@tamu.edu

**Keywords:** mass-rearing, animal waste, biodegradation, bioconversion

## Abstract

**Simple Summary:**

The black soldier fly (BSF) has gained a considerable amount of attention globally for its ability to convert organic material into valuable biomass for waste management and food and feed purposes. As the industry of producing BSF expands, connecting research and application is necessary to optimize mass-production facilities. Historically, research on the BSF has been conducted at a benchtop scale. Expanding these results to industrial practices is challenging, as results typically are not scalable. This study investigates the production of BSF fed animal manure (swine, dairy, and poultry) at a larger scale (thousands of larvae fed kg of diet) than what has been previously published (small-scale studies with hundreds of larvae fed g of diet).

**Abstract:**

Recent interest in the mass production of black soldier fly (BSF) larvae has resulted in many studies being generated. However, a majority of the studies are benchtop, or small-scale, experiments. Results generated from such studies may not translate to large-scale/industrial production. The current study was conducted at a conventional large-scale (10,000 larvae/treatment fed seven kg) to determine the impact on selected life-history traits when BSF were fed seven kg of manure (swine, dairy, or poultry) or a control diet (Gainesville diet: 50% wheat bran, 30% alfalfa meal, and 20% corn). Results showed larvae fed dairy manure took one to two days longer to develop to prepupation, with lower survivorship (45%) compared to those fed poultry or swine manure (>70%). Furthermore, the maximum larval weight was reached on day six for those fed swine manure, while other treatments achieved the maximum weight on day seven. However, larvae fed swine manure averaged 150 mg, while those fed the other diets ranged between 175 and 200 mg. Data from this study may be valuable for the industrialization of BSF. Companies using a scale varying from previously published work, including this study, should conduct pilot studies to optimize their system prior to implementation.

## 1. Introduction

Insects provide a variety of goods and services for human exploitation [1]. They may be reared for medicinal purposes, such as wound therapy [2], or to engineer antibodies [3] and vaccines [4]. They may be cultivated for other well-known purposes, such as honey [5], silk [6], and dye production [7], or can be used to control insect pest populations [8]. Insects may also be used for food and feed purposes [9]; however, this industry is still in its infancy in terms of operating on an industrial scale. Recent interest in exploiting insects in this manner has prompted numerous studies [10,11,12,13,14], which helps progress the idea and refine the industry; however, a majority of the published studies are based on small-scale (benchtop) experiments, which may not truly represent what occurs on a larger industrial scale. 

The black soldier fly (BSF), *Hermetia illucens* (L.), (Diptera: Stratiomyidae) has gained a considerable amount of attention. As discussed in previous publications, this species is distributed globally throughout temperate and tropical regions and is an ideal candidate for industrialization purposes, because it offers a means to manage a variety of wastes [15,16,17] and provides multiple revenue streams, such as the production of animal feed [9], biofuel [18,19], and fertilizer [20]. In systems using manure as a resource, the BSF reduces dry matter [21], pathogens [22,23], and odors [24]. However, most previously published work on BSF was performed on a small scale (e.g., several hundred larvae per replicate), which may not translate to an industrial scale. 

Methods used in small-scale studies are typically different than those employed by mass-production facilities (larval numbers in the thousands and fed kilograms of substrate rather than hundreds of larvae consuming grams over time). Both factors are known to impact development. For example, Banks et al. [25] showed that bulk feeding increased the development time and larval weight across three densities (1, 10, and 100 larvae) compared to those fed incrementally. Similarly, Barragán-Fonseca et al. [26] found that, with small-scale densities (50, 100, 200, or 400 larvae), an increased larval density lead to greater delays in development (up to 45 days) on low-nutrient diets but not on high-nutrient diets. Variations in the development time, larval and adult weights, and survivorship have also been reported across different larval densities (500–2000 larvae) of BSF fed the same diet [27]. Even the authors of the study being presented here have conducted such studies [28,29]. However, all of these studies are considered small-scale when compared to practices in the industry, and it is not known if similar results would occur on a larger scale.

Larval BSF density can hinder or, in some instances, enhance their performance. Bryant and Sokal [30] showed that low densities (80 eggs/18,000 mg of diet) and high densities (640 eggs/18,000 mg of diet) of house flies, *Musca domestica* L., (Diptera: Muscidae) faced different consequences during development. Low densities may result in poor conditioning of the diet (via metabolites produced by larvae), which impacts yeast growth and, ultimately, the availability of food [30]. However, an increased larval density may intensify the effects of competition, leading to reduced survivorship [30,31]. Larvae feed in aggregates generate heat [32], which, in turn, impacts BSF development and survivorship. Black soldier fly larvae reared at 30 °C developed the fastest (13 d), had the shortest prepupal development (8–10 d), and had the highest larval survivorship (90%) compared to those reared on temperatures that ranged from 10–42 °C [33]. Additionally, it is possible that higher densities produced more oral secretions (gut microbiota) that aided in the cooperative digestion of a resource [34]. As such, the larval density is a major factor that influences BSF performance.

The purpose of this study was to evaluate selected life-history traits of BSF fed swine, dairy, or poultry manure by using methods based on industrial standards [35]. Most of the data available on this species originates from small-scale studies, which may not translate to a larger production scale, as previously discussed. Results from this study may be valuable by providing a basis to compare findings from previous small-scale studies, as well as a paradigm to help optimize the mass-rearing conditions of BSF fed manure. 

## 2. Methods

### 2.1. Acquisition of BSF

Methods for this study were based on those conducted by Miranda et al. [29]. Black soldier flies were obtained from a colony that is maintained at the F.L.I.E.S. (Forensic Laboratory for Investigative Entomological Sciences) Facility at Texas A&M University in College Station, TX, USA. The colony originated from a colony maintained in Tifton, GA, USA and was maintained following modified methods proposed by Sheppard et al. [36].

### 2.2. Acquisition of Manure

Manure less than 12-h-old was used in this study. Swine manure was collected from a farm in Anderson, TX, USA, dairy manure was collected from a commercial dairy located in Stephenville, TX, USA, and poultry manure was collected from layer hens housed at the Poultry Science Research, Teaching and Extension Center at Texas A&M University, College Station, TX, USA. Manure was placed into 18.9 L buckets, covered with lids (Home Depot^®^, Leaktite™, Leominster, MA, USA), and transported to the F.L.I.E.S. Facility, where it was homogenized by hand-mixing for 5 min, transferred to 3.7 L Ziploc^®^ Freezer bags (S.C. Johnson & Son, Inc., Racine, WI, USA), and stored at −20°C until used. Before initiation of the experiment, the manure was allowed to thaw at room temperature for 24 h. Moisture contents of manure were measured gravimetrically with three 10 g samples following the methods described by Franson [37]. Initial moisture content for swine, dairy, and poultry manure were 72%, 83%, and 77%, respectively.

### 2.3. Experiment Design

Black soldier fly adults were maintained at the F.L.I.E.S. (Forensic Laboratory for Investigative Entomological Sciences) Facility in a 260 × 116 × 129 cm wooden cage lined with a fiberglass window screen (18 × 16 mesh size) in a greenhouse (25 °C, >50% relative humidity (RH)). To collect eggs, a 5.7 L Sterilite^®^ container (Sterilite^®^, Townsend, MA, USA) was filled with 500 g Gainesville diet (50% wheat bran, 30% alfalfa meal, and 20% corn meal) [38] saturated with RO (reverse osmosis) water (70%). Corrugated cardboard was cut into 5.0 × 2.0 × 0.5 cm pieces with five taped together to form a bundle, which was placed on the lid of the container described above. The lid of the container had a 15 × 7 cm hole cut in the center of the lid and was covered with wire mesh. The container remained in the wooden cage for 8 h, after which the cardboard was removed from the cage and placed in a 0.9 L Ball^®^ mason jar (Ball Corporation, Broomfield, CO, USA) covered with a paper towel, which was secured with the metal ring of the mason jar lid. The jar with the cardboard containing the eggs was stored in a Rheem Environmental Chamber (Asheville, NC, USA; 29 °C, 60% RH, and 16L:8D (light:dark)) until larvae were enclosed. Ten replicates of 100 newly-hatched larvae were hand counted and then weighed on an OHaus^®^ Adventure^™^ Pro AV64 balance (OHaus^®^ Corporation, Pine Brook, NJ, USA) to get the average weight of an individual larvae, which was then used to weigh approximately 10,000 larvae per replicate. Larvae were placed in 0.5 L deli food storage containers (Amazon.com Inc., Seattle, WA, USA) without a lid and were fed 150 g of Gainesville diet (70% moisture) for four days to decrease larval mortality prior to use in the experiment.

Treatments consisted of 10,000 4-d-old larvae fed 7 kg of swine, dairy, or poultry manure. Larvae in control groups were fed 7 kg of Gainesville diet (70% moisture). Larval diets were placed in the center of a 30 L Sterilite^®^ ClearView Latch^™^ storage container, with 700 g of dry Gainesville diet [38] placed around the perimeter of the wet diet to serve as a pupation substrate and to prevent developing larvae from escaping. Larvae were weighed prior to placement on the manure or Gainesville diets to determine that their weights were not significantly different. The deli containers with developing larvae were poured on top of the diets, and the containers (with the larvae and diets) were placed inside the environmental chamber (via complete randomized block design) described above. Three replicates of each treatment and control were used, and two trials were conducted.

Larvae were allowed to feed on the manure or Gainesville diets for two days prior to measuring the daily larval weight, as they were too small to find without significantly disturbing the media. The daily larval weight was measured by attempting to select 10 of the largest larvae for nine days, and a different set of larvae was selected each day. The development time to first prepupation was recorded upon observation of the first prepupa within a given container. On the day the first prepupa was observed, larvae were sifted from the media, and survivorship was calculated by dividing the total weight of all living larvae from each replicate by the average final larval weight. After the total larval weight was recorded, larvae were placed in 30 L Sterilite^®^ containers with 1 kg of dry Gainesville diet. The prepupal weight was measured by selecting 10 of the largest prepupae from each replicate when 40% reached the prepupal stage. All weights were measured using the balance described above.

### 2.4. Statistical Analyses

Larval weight, development time from placement on the manure to prepupation, percent prepupation (survivorship), and prepupal weight were analyzed across treatments and trials. An ANOVA was performed for each parameter listed. Statistics were performed using JMP^®^ PRO 14 (SAS Institute Inc., Cary, NC, USA). Normality was checked using a Shapiro-Wilk test, and equality of variance was checked using a Bartlett’s test. Tukey’s HSD (honest significant difference) was used for mean separation (*p* ≤ 0.05).

## 3. Results

### 3.1. Larval Weight

**Figure 1:**
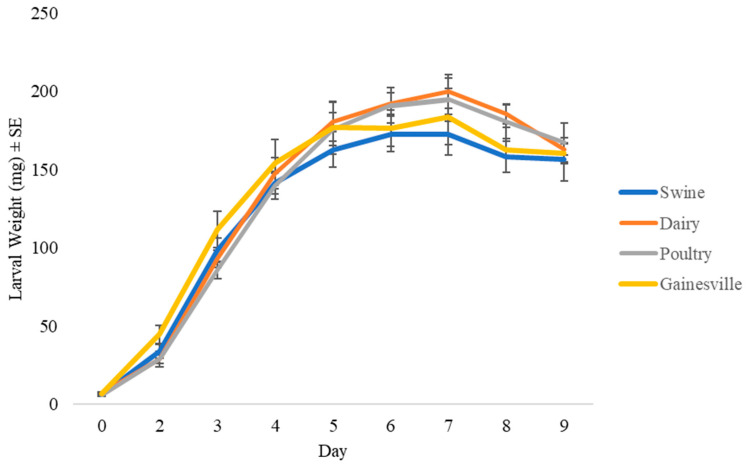
Mean larval weight (mg) ± SEM (^1^*n* = 6) of black soldier flies fed 7 kg of swine, dairy, or poultry manure or the Gainesville control diet [38] at 29 °C, 60% relative humidity (RH), and 16L:8D. ^1^*n* = number of replicates per treatment.

Larval weight did not differ significantly across larval diets (F_3,142_ = 1.9318; *p* = 0.1263). A significant trial effect was found (F_1,142_ = 163.6540; *p* < 0.0001), but no significant treatment by trial interaction was found (F_27,142_ = 0.7314; *p* = 0.8178). In general, individuals in trial two weighed 22% more than those in trial one. Larvae reared on swine manure reached a maximum weight on day six, while those fed the other diets did so on day seven. Furthermore, the maximum weight of larvae fed swine manure (150 mg) was less than those fed the other diets (175 to 200 mg) (Figure 1).

### 3.2. Development Time to First Prepupation

**Figure 2:**
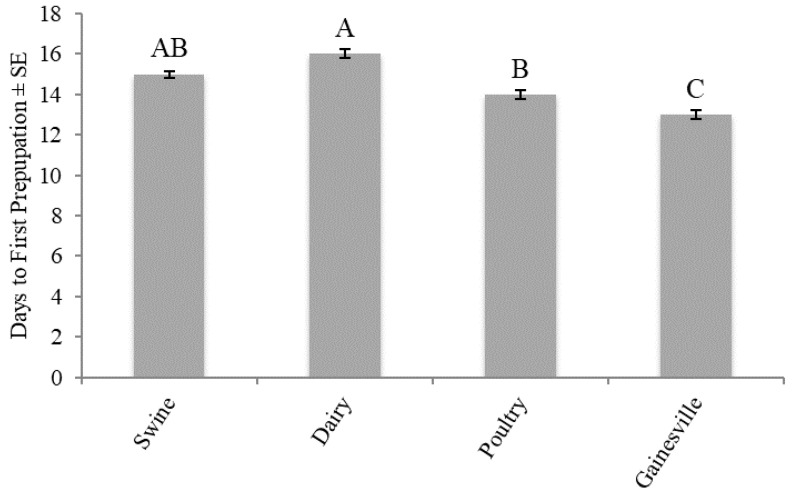
Development time (d) to first prepupation (mean ± SE, ^1^n = 6) of black soldier fly larvae fed 7 kg of swine, dairy, or poultry manure or the Gainesville control diet [38] at 29 °C, 60% RH, and 16L:8D. Different letters (A–C) indicate significant differences between treatments (α = 0.05) and ANOVA, followed by Tukey’s HSD. ^1^n = number of replicates per treatment.

Development time to first prepupation differed significantly (F_3,16_ = 16.9048; *p* < 0.001) across the larval diets. No trial effect (F_1,16_ = 0.1429; *p* = 0.7104) or treatment by trial interaction (F_3,16_ = 0.5238; *p* = 0.6721) was found. The shortest development time was found for those fed the Gainesville diet (13 d). In regard to those fed manure, the shortest development time was found for those fed poultry (14 d) and swine manures (15 d), whereas those fed dairy manure took an additional day (16 d) to reach prepupation (Figure 2). 

### 3.3. Percent Prepupation

**Figure 3:**
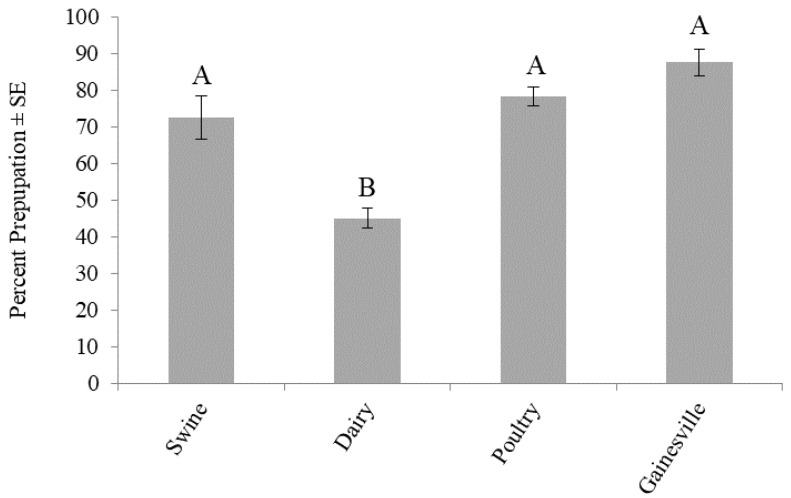
Percent prepupation (mean ± SE, ^1^*n* = 6) of black soldier fly larvae fed 7 kg of swine, dairy, or poultry manure or the Gainesville control diet [38] at 29 °C, 60% RH, and 16L:8D. Different letters (A, B) indicate significant differences between treatments (α = 0.05) and ANOVA, followed by Tukey’s HSD. ^1^*n* = number of replicates per treatment.

Survivorship to prepupation was significantly different (F_3,16_ = 22.2899; *p* < 0.0001) across the larval diets. No trial effect (F_1,16_ = 0.6001; *p* = 0.4498) or treatment by trial interaction (F_3,16_ = 1.2535; *p* = 0.3235) was found. The highest percent prepupation across all diets was found for those fed the Gainesville diet (88%). Survivorship to prepupation was significantly lower in dairy manure (45%), but not in poultry (78%) or swine (73%) manures, relative to the Gainesville diet (Figure 3). 

### 3.4. Prepupal Weight

**Figure 4:**
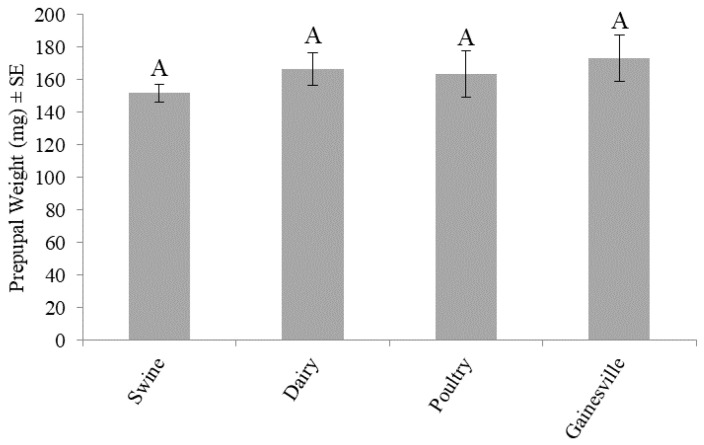
Prepupal weight (mg) (mean ± SE, ^1^*n* = 6) of black soldier fly larvae fed 7 kg of swine, dairy, or poultry manure or the Gainesville control diet [38] at 29 °C, 60% RH, and 16L:8D. Different letters (A) indicate significant differences between treatments (α = 0.05) and ANOVA, followed by Tukey’s HSD. ^1^*n* = number of replicates per treatment.

Prepupal weight did not differ significantly (F_3,16_ = 0.5997; *p* = 0.6245) across the larval diets. No trial effect (F_1,16_ = 3.5717; *p* = 0.0770) or treatment by trial interaction (F_3,16_ = 0.0837; *p* = 0.9679) was observed. Larvae fed the Gainesville diet weighed approximately 173 mg, and those fed poultry, swine, and dairy weighed 163, 152, and 167 mg, respectively (Figure 4).

## 4. Discussion

The results from this study demonstrate that diet can impact the production of BSF. Larvae fed the Gainesville diet [38] had the shortest development time (13 d) (Figure 2), the highest survivorship (88%) (Figure 3), and produced the heaviest prepupae (173 mg) (Figure 4). In regard to those fed manure, variations in development time and survivorship were found across manure types. Although larval (Figure 1) or prepupal weights (Figure 2) did not differ across treatments (*p* < 0.05), a longer development time (one to two days) was required for larvae to reach the prepupal stage, and fewer larvae survived (45%) when fed dairy manure. Similarly, in regard to the maximum weight, those fed swine manure reached their peak weight one day earlier than those fed dairy or poultry manures but weighed 25–50 mg less. These differences are important in respect to production efficiencies and bioconversion and may be explained by variations in the manure types. 

Manure from different animals varies in chemical and physical compositions [39]. Poultry manure is typically higher in nutrients and lower in fiber than dairy manure [40], and these differences may explain differences in development and survivorship in the current study. Variation in development time and survivorship could translate to variation in production efficiency and bioconversion yields. Although prepupal weights were not significantly different across the treatments (Figure 4), slower developments for those fed dairy manure (Figure 2) and lower survivorship (Figure 3) may have implications in higher costs of rearing (longer development time) and lower revenue (less yield). It is possible that those fed dairy manure were able to reach similar prepupal weights to those fed swine or poultry manures because the larvae were fed on the resource longer and were subject to reduced intraspecific competition because of high mortality. Additionally, BSF are thought to be generalist feeders, capable of digesting various wastes [15,17,41]. Compared to house flies, BSF have a larger arsenal of digestive enzymes that helps them acquire adequate nutrition from a variety of substrates to sustain their adult stage [41]. It is possible that the larval and prepupal weights did not differ significantly because BSF have such a wide range of digestive enzymes. 

Previous studies that focus on BSF fed manure have been performed on a smaller scale (i.e., lower number of larvae per replicate) than the current study. Myers et al. [21] were the first to examine BSF development on dairy manure. Specifically, this study fed three hundred four-day-old larvae 27, 45, 54, or 70 g of dairy manure daily and found that the larval development to prepupation was significantly different (*p* ≤ 0.05) between the lower feed ration and the other rations and lasted 26–30 d, which was up to two weeks longer than the findings from the current study (16 d). Survivorship to prepupation was not significantly different across the treatments for the aforementioned study (71–77%) but was greater than the survivorship found in the current study (45%). The prepupal weight was significantly different (*p* ≤ 0.05) across the treatments in the small-scale dairy study (89–137 mg), and this was less than that observed in the current study (167 mg). However, when comparing the current study to another small-scale study that used a different manure type, such as poultry manure, impacts on the prepupal weight and development time were similar, but survivorship differed across the scales. For example, Lalander et al. [17], fed two hundred 10-d-old larvae poultry manure (40 mg DM/larva/d) and found prepupae weighed 165 mg, and those from the current study weighed 163 mg with the same development time to prepupation from the placement on manure (14 d). However, the current study found a lower survivorship (78% in the current study vs. 92% in Lalander et al. [17]). Yet, when we examine findings from our own small-scale study [29], which used larvae that were derived from the same colony as the current study, less time was needed for development in the small-scale study for those fed poultry manure (11 d vs. 14 d in the large-scale study), but more time was required in the small-scale study for those fed swine manure (17 d vs. 15 d in the current study), and larvae fed dairy manure required 16 d to develop, regardless of the scale. Although the percent prepupation was similar for those fed swine manure (84% vs. 73% in the current study), differences were more obvious for those fed dairy (93% vs. 45% in the current study) or poultry manures (78% vs. 95% in the current study). Additionally, differences in prepupal weight across the scales are apparent for swine, dairy, and poultry manures, as those in the small-scale study weighed 83, 99, and 114 mg compared to 152, 167, and 163 mg, respectively, in the current study. 

Based on a comparison between this study and others conducted at a smaller scale, scale likely impacts the production of the BSF. Despite the fact that some aspects of the methodologies from some of the small-scale studies differed (such as rearing conditions and age of larvae at the initiation of the experiment) from the current study, there is overlap in methodologies among the studies that helps eliminate these factors from consideration as potential reasons for variation across the scales. For example, the rearing temperature for Myers et al. [21] (27 °C), and Lalander et al. [17] (28 °C) differed from the current study (29 °C); however, it is unlikely that this factor is largely responsible for differences across the scales, as Miranda et al. [29] used the same rearing temperature described by the current study, and differences in the development time, survivorship, and weight were found. The relative humidity should also not be considered influential, as most studies reared larvae at or around 60% RH. Additionally, although the age of the larvae at the initiation of the experiment differed between Lalander et al. [17] and the current study, it is also unlikely that this factor is responsible for the variations among the studies, because Myers et al. [21], Miranda et al. [29], and the current study conditioned larvae on the same diet (Gainesville diet [38]) for the same amount of time (four days) prior to the initiation of the experiment, and differences were found between these studies as well. 

Interestingly, diet compositions across the manure types likely impacts the variation across the scales. For example, the development time did not differ for larvae fed dairy manure between Miranda et al. [29] and the current study, but differed for those fed poultry manure. Yet, when we compared the findings from Myers et al. [21] for dairy manure and those from Lalander et al. [17] for poultry manure to the current study, we saw that the opposite occurred, with delayed development reported by Myers et al. [21] for those fed dairy manure (26–30 d vs. 16 d) and the same development time as the current study for those fed poultry manure by Lalander et al. [17] (14 d). As previously discussed, manure types vary in chemical and physical properties and can vary within the same type, which may explain the differences observed between our study and previous small-scale studies. The development time is influenced by weight and survivorship, and so, the diet composition impacts those parameters as well. Furthermore, we find when comparing our study to Miranda et al. [29], which used larvae from the same colony with the same rearing conditions, and manure collected from the same facilities as the current study; differences across the manure types for development time, prepupal weight, and survivorship do not follow the same pattern across the scales. For example, for development time, the results were similar regardless of the scale, with swine and dairy not significantly different (*p* ≤ 0.05) from each other but significantly different from poultry manure in either study. Larvae from the small-scale experiment developed faster on poultry manure (by three days) than those in the large-scale study, and these findings correlate with those from Barragán-Fonseca et al. [26], which found that lower densities and higher nutrient concentrations accelerated development. However, the pattern of differences across manure types for prepupal weights differed across the scales, with more pronounced differences among the manure types at the smaller scale compared to the larger scale. Specifically, larvae fed swine manure in the small-scale study were significantly different (*p* ≤ 0.0001) in regard to prepupal weights from those fed poultry manure, but larvae fed swine, dairy, or poultry manure in the large-scale study were not significantly different (*p* = 0.0624). Barragán-Fonseca et al. [26] found that the weights increased with the increased nutrient concentrations, and this was the case for the small-scale study but not for the large-scale study, as those fed dairy manure weighed more (167 mg) than those fed poultry (163 mg) or swine (152 mg). Although dairy manure is generally considered a lower-quality manure compared to swine or poultry manure, higher prepupal weights for larvae fed dairy manure may be explained by the lower survivorship, as previously discussed. Finally, the pattern of differences across manure types for percent of pupation differed across the scales, with more pronounced differences on the larger scale (*p* ≤ 0.0001) than in the smaller scale (*p* = 0.0004), but the findings from our studies do not agree with Barragán-Fonseca et al. [26] that survivorship increases with higher densities, as fewer individuals were produced from the large-scale study (45–78% survivorship) compared to the small-scale study (84–95% survivorship); however, our findings do agree that survivorship increases with increasing nutrient concentrations. The extent of the impact of diet compositions on differences across the scales is poorly understood due to a lack of large-scale studies; therefore, small-scale studies should include a large-scale component when possible. 

Other factors known to influence BSF development are moisture [28,42,43], pH [44,45], species strain [46], and size of the rearing container [47]. However, because most studies are conducted with a few-hundred larvae, the impact of these factors is not necessarily the same at higher scales. It may thus be difficult for individuals interested in mass-producing BSF to extrapolate and apply information with expectations similar to those reported from studies that use methods that differ from industrial standards. It should be noted that the authors of this paper have also performed small-scale studies [28,29] but recognize that the outcomes of these studies may differ at a larger scale. Additionally, there are facilities that produce larvae at higher densities than 10,000 larvae and provide larvae more than 7 kg of diet, and so it is possible that the results from this study may not translate on a higher production scale. For these reasons, future studies should investigate BSF life-history parameters at larger scales than the current study. 

## 5. Conclusions

The current study shows differences in the development time and survivorship for larvae fed different manure types. Although no significant difference was found across the manure types for prepupal weight, those provided dairy manure took one to two days longer to develop, with fewer individuals surviving to the prepupal stage (45% vs. >70%) when compared to those provided swine or poultry manures. Additionally, this study highlights the potential differences likely to exist across the production scales and urges future studies to perform their work on larger scales to advance the industrialization of BSF.
